# Visual prognosis better in eyes with less severe reduction of visual acuity one year after onset of Leber hereditary optic neuropathy caused by the 11,778 mutation

**DOI:** 10.1186/s12886-017-0583-3

**Published:** 2017-10-18

**Authors:** Yukihiko Mashima, Kazuteru Kigasawa, Kei Shinoda, Masato Wakakura, Yoshihisa Oguchi

**Affiliations:** 1 Department of Ophthalmology, Keio University School of Medicine, 35 Sninanomachi, Shinjuku-ku, Tokyo, 160-8582 Japan; 2 Kyorin Eye Center, Kyorin University School of Medicine, 6-20-2 Shinkawa, Mitaka-shi, Tokyo, 181-8611 Japan; 3 Department of Ophthalmology, Faculty of Medicine, Saitama Medical University, Iruma-gun, Saitama, 350-0495 Japan; 4 Inouye Eye Hospital, 4-3 Kandasurugadai, Chiyoda-ku, Tokyo, 101-0062 Japan

**Keywords:** Leber hereditary optic neuropathy, Visual recovery, Lowest visual acuity, 11,778 mutation, Japanese

## Abstract

**Background:**

Patients with Leber hereditary optic neuropathy (LHON) have a progressive decrease of their visual acuity which can deteriorate to <0.1. Some patients can have a partial recovery of their vision in one or both eyes. One prognostic factor associated with a recovery of vision is an early-age onset. The purpose of this study was to determine other clinical factors that are predictive of a good visual recovery.

**Methods:**

Sixty-one Japanese LHON patients, with the 11,778 mutation and a mean age of 23.1 ± 12.1 years at the onset, were studied. All patients were initially examined at an acute stage of LHON and were followed for 3 to 10 years. At 1 year after the onset, the lowest visual acuity was <0.1 in all eyes. We studied the following parameters of patients with/without a final visual acuity of ≥ 0.2: sex; heavy consumption of cigarettes and alcohol; taking idebenone; mean age at onset; mean lowest visual acuity; and distribution of the lowest and the final visual acuity.

**Results:**

Fifteen (24.6%) of the 61 patients or 25 (20.5%) of the 122 eyes had a recovery of their visual acuity to ≥ 0.2. The mean age at onset of these 15 patients with visual recovery to ≥ 0.2 was 17.5 ± 7.7 years, and that of the 46 patients without visual recovery to ≥ 0.2 was 25.0 ± 12.8 years (*P* = 0.02, Mann-Whitney *U* test). The mean lowest visual acuity of the 25 eyes with visual recovery ≥ 0.2 was 0.04, and that of the 97 eyes without visual recovery to ≥ 0.2 was 0.015 (*P* < 0.001, Mann-Whitney *U* test). Fifty percent (15/30) of the eyes whose lowest visual acuity was ≥ 0.04 during 1 year after the onset had a visual recovery to ≥ 0.2, while 11% (10/92) of the eyes whose the lowest visual acuity was ≤ 0.03 had a visual recovery to ≥ 0.2 (*P* < 0.001, *χ* ^2^ test). There were no significant differences in the other clinical factors.

**Conclusion:**

A final visual acuity of ≥ 0.2 was associated with a less severe reduction of the visual acuity at 1 year after the onset. Our findings can be used to predict the visual prognosis in LHON patients.

## Background

Leber hereditary optic neuropathy (LHON, OMIM 535000) is a mitochondrial DNA (mt DNA)-related disorder with a prevalence of 1:31,000 to 1:50,000 [1, 2]. Patients with LHON are usually young men who have an acute or subacute progressive decrease in their decimal visual acuity to <0.1. Within a year after the onset, the patients have a pallor of the optic disc and suffer from a severe reduction of vision due to the degeneration of the retinal ganglion cells and their axons. However, even after the reduction of the central vision for months to years, some patients can have a partial recovery of their central vision in one or both eyes with an opening of a few degrees within the central scotoma, i.e., a fenestrated scotoma [3].

More than 90% of the LHON patients carry one of three mutations at nucleotide positions 3460 (np3460G > A), 11,778 (np11778G > A), or 14,484 (np14484T > C) of the mt DNA [4–6]. The major difference among these patients is the visual prognosis. The 11,778 mutation has the poorest prognosis with partial visual recovery rates from 4 to 25% [5, 7–11], and the 14,484 mutation has the best prognosis with partial visual recovery rates from 37 to 70% [5, 8, 9, 11, 12]. The visual recovery of the patients with the 3460 mutation has an intermediate rate of 15 to 40% [5, 10, 11, 13]. Legal blindness in the USA is defined as a corrected visual acuity of ≤ 20/200 (decimal visual acuity 0.1) with limited vision and an inability of distinguishing details, reading, or doing fine work. In Japan, a clinical study of LHON patients with the 11,778 mutation previously reported that 15 of 88 patients (17.0%), or 22 of 176 eyes (12.5%) had a recovery of vision to ≥ 0.2 (20/100) [14]. The patients with good visual recovery were reported to have a lower teenage onset among the patients with the 11,778 [10, 15–19] or the 14,484 mutation [8, 9, 12]. Thus, a spontaneous visual recovery appears to be more likely when the visual reduction begins at a younger age.

Idebenone (Raxone®) was approved for LHON patients in Europe in September 2015, and the clinical trial demonstrated that the vision in some patients at an early stage of the disease improved with megadose (900 mg/day) of idebenone [20, 21]. The purpose of this study was to determine factors that are predictive of a good visual recovery, other than a younger age at onset. To accomplish this, we examined the relationship between the final visual acuity and the lowest visual acuity during the 1-year period after the onset, sex, heavy consumption of cigarettes smoking and alcohol, or taking idebenone. We studied 61 Japanese LHON patients with the 11,778 mutation and measured their visual acuities intermittently for 3 to 10 years after the onset. The results indicated that a final visual acuity of ≥ 0.2 was associated with a less severe reduction in the visual acuity during the 1-year period after the onset.

## Methods

### Patients

Sixty-one Japanese LHON patients, 56 men and 5 women, with the 11,778 mutation were studied. The clinical data relevant to this study such as sex, age at onset, family history, environmental factors, visual acuity, primary LHON mutation, and treatment were obtained through the medical records of the patients. From 1980, 54 patients were referred and followed at the Neuro-ophthalmology Clinic of Keio University Hospital (Tokyo, Japan). After 2005, seven patients were referred and followed at the Kamoshita-Eye Clinic (Tokyo, Japan). After 1990, DNA analysis was mostly performed at the Keio University Hospital by the PCR-Restrict Enzyme method, and some of the patients were genetically analyzed before visiting the Keio University Hospital or Kamoshita-Eye Clinic by commercially available methods.

All of the patients first visited the clinics at an acute or a subacute stage of the disease process. Their mean age at the onset was 23.1 ± 12.1 years with a range of 9 to 65 years and a median age of 18 years. Within 1 year after the onset of the disease, the lowest visual acuity was <0.1, and it was associated with central scotomas by Goldmann perimetry in all eyes.

Since 1990, 21 of the 61 patients received 90 mg/day of idebenone (Avan®, Takeda Pharmaceutical Co Ltd., Japan), 120 mg/day of riboflavin, and 750 mg/day of ascorbic acid after giving their informed consent. All agents were taken orally for about 1 year after the onset of the disease. Since 1994, 17 of these 21 patients who took idebenone (Avan®) also received topical 0.12% isopropyl unoprostone (Rescula®, Fujisawa Pharmaceutical Co Ltd., Japan, now Santen Pharmaceutical Co Ltd., Japan) daily in each eye [22] to lower the intraocular pressure and to improve circulation of the optic disc [23]. This was done off-label but after receiving their informed consent. Isopropyl unoprostone has been approved for glaucoma and ocular hypertension. Isopropyl unoprostone has been shown to be a big potassium (BK) channel opener [24]. Six of the 61 patients received riboflavin and ascorbic acid. Idebenone (Avan®) was withdrawn from the market in Japan in 1998. After that, instead of idebenone, commercially available non-prescribed coenzyme Q10 (100 mg/day), riboflavin and ascorbic acid were taken by 7 of the 61 patients. Twenty-seven of the 61 patients received no treatment.

Six patients were heavy consumers of cigarettes and alcohol at the time of this study. The age at the onset of the disease was 22, 27, 35, 36, 45, and 49 years old, respectively. All patients were prohibited from smoking and drinking any alcohol after being diagnosed with LHON.

A written informed consent was obtained from patients for the DNA tests, the taking of idebenone (Avan®), vitamins or unopurostone eye drops, and publications of data including case studies. The procedures used in this study were approved by the Ethics Committee of Keio University Hospital. The visual fields were determined by Goldmann perimetry, and the best-corrected decimal visual acuity (BCVA) was measure with a Landolt ring visual acuity chart. We retrospectively analyzed the BCVA in the 61 patients from the initial acute stage and followed for 3 to 10 years. The BCVA was measured every 3 months during the 1 year period after the onset, and then, every 6 months.

### Statistical analyses

The mean age at onset in the patients with a final visual acuity of ≥ 0.2 was compared to that in the patients without a final visual acuity ≥ 0.2 by non-parametric Mann-Whitney *U* tests. The decimal visual acuity was converted to the logarithm of the minimum angle of resolution (logMAR) for the statistical analyses. The mean lowest visual acuity in the eyes with and without a final visual acuity of ≥ 0.2 was compared by non-parametric Mann-Whitney *U* tests. Differences in the number of eyes with a final visual acuity of ≥ 0.2 were evaluated according to lowest visual acuity in each eye during the 1-year period after the onset, sex distribution, history of heavy consumption of cigarettes and alcohol, or taking idebenone by *χ* ^2^ tests. All of the results are expressed as the means ± standard deviations, and *P* values <0.05 were considered statistically significant (SPSS Statistics Ver.19, IBM, Japan).

## Results

**Fig. 1:**
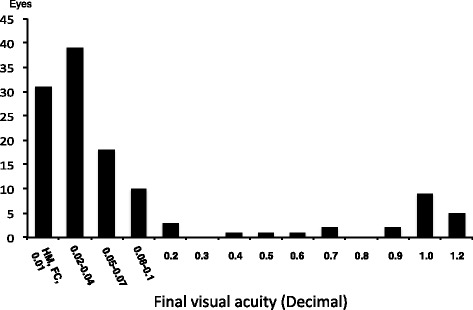
Distribution of the final visual acuities in 122 eyes of 61 patients with LHON. Legal blindness is defined in the USA as a visual acuity of ≤ 0.1. Twenty-five eyes of 122 eyes (20.5%) had a final visual acuity of ≥ 0.2. HM: hand motion, FC: finger count

Twenty-five of the 122 eyes (20.5%) or 15 of the 61 LHON patients (24.6%) had a recovery of their visual acuity to ≥ 0.2 (Fig. 1), and 12 (19.7%) of the 61 patients or 21 (17.2%) of the 122 eyes had a recovery of their visual acuity to ≥ 0.5. Forty-six of the 61 patients (75.4%) were legally blind by the USA definition as a visual acuity of ≤ 0.1. The mean age at onset was 17.5 ± 7.7 years in the 15 patients with a final visual acuity of ≥ 0.2 in at least one eye, and 25.0 ± 12.8 years in the 46 patients with a final visual recover of ≤ 0.1 in both eyes. Thus, the mean age at onset of patients who had better visual acuity at a later stage was significantly younger than the age of patients who had poorer visual acuity of legal blindness (*P* = 0.02, Mann-Whitney *U* test).

**Fig. 2:**
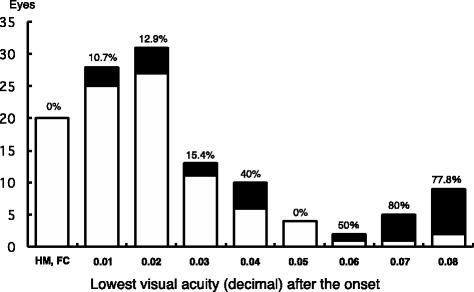
Distribution of the lowest visual acuity at one year after the onset of LHON in 122 eyes of 61 patients with LHON. The black areas (25 eyes) indicate a final visual acuity of ≥ 0.2, and the white areas (97 eyes) a final visual acuity of ≤ 0.1. Fifty percent of the eyes whose visual acuity at the lowest point was 0.06, 80% of the eyes with the lowest visual acuity was 0.07, and 77.8% of the eyes with the lowest visual acuity of 0.08 recovered vision to ≥ 0.2. HM: hand motion, FC: finger count

The distribution of the visual acuities of the eyes with the lowest visual acuity during the 1-year period after the onset with and without treatments is shown in Fig. 2. The black areas (25 eyes) represent eyes with a final visual acuity of ≥ 0.2, and the white areas (97 eyes) represent eyes with a final visual acuity of ≤ 0.1. Fifty percent of the eyes that had a lowest visual acuity of 0.06, 80% of the eyes that had a lowest visual acuity of 0.07, and 77.8% of the eyes that had a lowest visual acuity of 0.08 recovered vision to ≥ 0.2. The mean lowest decimal visual acuity within 1 year after the onset among the 25 eyes with a visual recovery of ≥ 0.2 was 0.04 (1.41 ± 0.32 logMAR units), while that among the 97 eyes with a final visual acuity of ≤ 0.1 was 0.015 (1.82 ± 0.33 logMAR units). There was a significant difference in the lowest visual acuity during the 1-year period between the eyes with and without visual recovery to ≥ 0.2 (*P* < 0.001, Mann-Whitney *U* test). The eyes were divided into four groups based on whether the final visual acuity was or was not ≥ 0.2, and whether the worst visual acuity was or was not ≥ 0.04. Fifty percent (15/30) of the eyes with the lowest visual acuity of ≥ 0.04 had a visual recovery to ≥ 0.2. On the other hand, about 11% (10/92) of the eyes with the lowest visual acuity of ≤ 0.03 had a visual recovery to ≥ 0.2 (*P* < 0.001, *χ* ^2^ test) (Table 1). In addition, 55% (11/20) or 70% (11/16) of the eyes with the lowest visual acuity of ≥ 0.05 or 0.06 had a visual recovery to ≥ 0.2, respectively (*P* < 0.001, *χ* ^2^ test).

**Table 1:** Final visual acuity of 0.2 or better related to lowest visual acuity

	Lowest visual acuity ≤ 0.03	Lowest visual acuity ≥ 0.04
Final visual acuity ≥ 0.2	10 eyes	15
Final visual acuity ≤ 0.1^a^	82	15

^a^Legal blindness in USA

Seven of the 21 patients who received 90 mg/day of idebenone (Avan®) had a final visual acuity of ≥ 0.2, while eight of the 40 patients who did not received idebenone had a final visual acuity of ≥ 0.2. There was no significant difference in the final visual acuity of ≥ 0.2 (*P* > 0.05. *χ* ^2^ test). One of the 6 patients with heavy consumption of cigarettes and alcohol had a final visual acuity of ≥ 0.2, while 14 of the 55 patients without heavy consumption had a final visual acuity of ≥ 0.2. There was no significant difference in the final visual acuity of ≥ 0.2 (*P* > 0.05. *χ* ^2^ test). One of the 5 female patients had a final visual acuity of ≥ 0.2, while 14 of the 42 male patients had a final visual acuity of ≥ 0.2. There was no significant difference in the final visual acuity of ≥ 0.2 between the men and women (*P* > 0.05. *χ* ^2^ test).

**Fig. 3:**
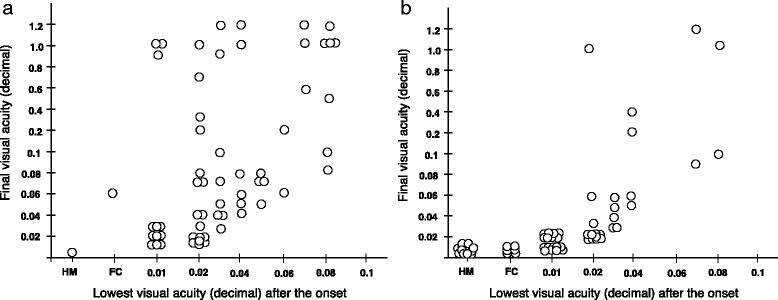
Relationship between the lowest visual acuity during the one year of the onset of LHON and the final visual acuity. **a** Sixty-two eyes of 31 patients with age at onset of LHON ≤ 18 years. **b** Sixty eyes of 30 patients with age at onset of LHON of ≥ 19 years. HM: hand motion, FC: finger count

The mean age at onset was 17.5 ± 7.7 years in the 15 patients with a final visual acuity of ≥ 0.2, and the median age at the onset in the 61 patients was 18 years with a range of 9 to 65 years. Visual recovery in patients with the 11,778 mutation was more likely when the visual reduction begins at a younger age [10, 15–19]. Thus, we divided the 61 patients into the two groups by the median age of 18 years: the 31 patients with a median age of ≤ 18 years and the 30 patients with a median age of ≥ 19 years. The relationship between the lowest visual acuity within the 1-year period after the onset and the final visual acuity in these two groups is shown in Fig. 3. Although there were more patients who recovered their visions to ≥ 0.2 in the eyes with an age at onset of ≤ 18 years (Fig. 3a) than those with an onset of ≥ 19 years (Fig. 3b), the ratio of such patients increased when their visual acuity at the lowest value was over 0.04 for both age groups.

## Discussion

Patients with LHON generally have a significant reduction of vision, and the BCVA in the majority of the patients is <0.1. A spontaneous partial recovery has also been reported in 4 to 70% of the eyes with the rates dependent on the causative mutation. The factors that are predictive of a good recovery of vision are an early age of onset [8, 9, 12, 15–19], slow progressive course [10, 11, 25], caused by the 14,484 mutation [5, 8, 9, 11, 12], thicker retinal nerve fiber layer by optical coherence tomography (OCT) [25, 26], and larger vertical diameter of the optic disc in the OCT images [27]. Children generally have a slower progression of the disease process [10, 11, 25].

Fifteen (24.6%) of the 61 patients or 25 (20.5%) of 122 eyes had a final visual acuity of ≥ 0.2, and 12 (19.7%) of the 61 patients, or 21 (17.2%) of the 122 eyes had a recovery of their visual acuity to ≥ 0.5. On the other hand, only 4% of the patients with the 11,778 mutation in the USA and 9% of the patients in Europe had a visual recovery to ≥ 0.5 [7, 8]. The Japanese patients with the 11,778 mutation had a better visual recovery. Hotta et al. [14] reported that average progression of visual loss was 6.2 months in the 89 Japanese patients, and 3.7 months in the 72 patients in the USA. Slowly progressive course after the onset in Japanese LHON patients would have better visual outcome [10, 11, 25]. MtDNA background of 11,778/haplotypes in LHON patients could affect the clinical expression and visual loss especially in patients with European-specific haplotype J [28, 29]. In Asian LHON patients, the 11,778/haplotype M is a major group and could affect the clinical expression [30]. Although recent studies have shown that the background of haplotypes could affect the clinical expression, their association with visual prognosis in LHON patients is unknown. Different mtDNA background 11,778/haplotypes might be related to slow progressive course after the onset and the final visual acuity. Further studies will be needed to determine whether a significant association between 11,778/haplotypes and visual prognosis is present.

We defined a good visual recovery as a final decimal visual acuity of ≥ 0.2 because the lowest visual acuity in all eyes was <0.1 within 1-year after the onset of LHON, and legal blindness has been defined as a visual acuity of ≤ 0.1 in the USA. In our earlier publication including some of the 15 patients in this study, most of the patients who had a visual recovery to ≥ 0.2 had stable or relatively stable fixation confirmed by a static fundus-microperimetry [31]. The patients with better visual recovery and stable fixation reportedly had areas of improved retinal sensitivity within the central scotoma [31–33].

The 15 patients with a final visual acuity of ≥ 0.2 had two characteristics. First, the mean age of the disease at onset was significantly younger than those with a visual recovery to ≤ 0.1 (17.5 ± 7.7 years vs 25.0 ± 12.8 years). This is consistent with previous reports of the patients with the 11,778 mutation [10, 15–19]. Second, 50% (15/30), 55% (11/20), or 70% (11/16) of the eyes with the lowest visual acuity of ≥ 0.04, 0.05, or 0.06 during the 1-year period after the onset had a visual recovery to ≥ 0.2. Although the number of the patients whose vision recovered to ≥ 0.2 was higher in the 31 patients who were ≤ 18 years than in the 30 patients who were ≥ 19 years, this favorable visual recovery in the patients with the lowest visual acuity of ≥ 0.04 was found in both age groups. In a case report, some patients who were ≥ 30 years at the onset of the disease and with the 11,778 mutation were reported to have good visual recovery when they experienced a less severe reduction of the visual acuity of 0.05 to 0.08 after the onset [34–37].

The dose of idebenone (Avan®) was 90 mg/day, which is one-tenth the dose of 900 mg/day of idebenone (Raxone®) in the clinical trial in Europe [20, 21]. In our previous small case study with 90 mg/day of idebenone (Avan®), there was no significant difference in number of the eyes with visual recovery to ≥ 0.3 in the patients with/without idebenone (Avan®) [22]. Because our therapeutic interventions such as idebenone (90 mg/day), coenzyme Q10 (100 mg/day), and vitamins were low-doses, it is not certain whether the relationship between the lowest visual acuity and final visual acuity represented the natural course or the therapeutic intervention. However, the lowest visual acuity of ≥ 0.04 during 1-year period after the onset could be one of the favorable prognostic factors for the recovery of vision in the patients with the 11,778 mutation with or without any therapeutic interventions.

## Conclusions

In the early phase of LHON patients, the factors that are predictive of a recovery of vision are recognized to be an early age of onset [8, 9, 12, 15–19] and type of primary mtDNA mutations [3]. Patients with LHON caused by the 11,778 mutation have the poorest prognosis with low partial visual recovery rates. Of our 61 Japanese patients with the 11,778 mutation, 24.6% (15/61) had a visual recovery to ≥ 0.2 in one or both eyes. A final visual acuity of ≥ 0.2 was associated with a less severe reduction of the visual acuity during the 1-year period after the onset as well as being younger at the onset of the disease. These findings could be useful in determining the visual prognosis of LHON patients when conducting therapeutic studies. As this cohort study was retrospective from the point of the final visual acuity, prospective studies of the relationship between the lowest visual acuity after the onset of the disease and the recovery of vision with/without therapeutic intervention are necessary to confirm our results.

## Abbreviations

BCVA: Best-corrected visual acuity
FC: Finger count
HM: Hand motion
LHON: Leber hereditary optic neuropathy
logMAR: logarithm of the minimum angle of resolution
mtDNA: mitochondrial DNA
OCT: Optical coherence tomography
